# Deciphering exciton-generation processes in quantum-dot electroluminescence

**DOI:** 10.1038/s41467-020-15944-z

**Published:** 2020-05-08

**Authors:** Yunzhou Deng, Xing Lin, Wei Fang, Dawei Di, Linjun Wang, Richard H. Friend, Xiaogang Peng, Yizheng Jin

**Affiliations:** 1 Center for Chemistry of High-Performance & Novel Materials, State Key Laboratory of Silicon Materials, Department of Chemistry, Zhejiang University, 310027 Hangzhou, China; 2 Center for Chemistry of High-Performance & Novel Materials, Department of Chemistry, Zhejiang University, 310027 Hangzhou, China; 3 State Key Laboratory of Modern Optical Instrumentation, College of Optical Science and Engineering, Zhejiang University, 310027 Hangzhou, China; 4 State Key Laboratory of Modern Optical Instrumentation, College of Optical Science and Engineering, International Research Center for Advanced Photonics, Zhejiang University, 310027 Hangzhou, China; 5 Cavendish Laboratory, Department of Physics, University of Cambridge, Cambridge, CB3 0HE UK

**Keywords:** Nanoscale materials, Lasers, LEDs and light sources

## Abstract

Electroluminescence of colloidal nanocrystals promises a new generation of high-performance and solution-processable light-emitting diodes. The operation of nanocrystal-based light-emitting diodes relies on the radiative recombination of electrically generated excitons. However, a fundamental question—how excitons are electrically generated in individual nanocrystals—remains unanswered. Here, we reveal a nanoscopic mechanism of sequential electron-hole injection for exciton generation in nanocrystal-based electroluminescent devices. To decipher the corresponding elementary processes, we develop electrically-pumped single-nanocrystal spectroscopy. While hole injection into neutral quantum dots is generally considered to be inefficient, we find that the intermediate negatively charged state of quantum dots triggers confinement-enhanced Coulomb interactions, which simultaneously accelerate hole injection and hinder excessive electron injection. In-situ/operando spectroscopy on state-of-the-art quantum-dot light-emitting diodes demonstrates that exciton generation at the ensemble level is consistent with the charge-confinement-enhanced sequential electron-hole injection mechanism probed at the single-nanocrystal level. Our findings provide a universal mechanism for enhancing charge balance in nanocrystal-based electroluminescent devices.

## Abstract (web summary)

Today it remains unclear how excitons are electrically generated in individual nanocrystals. Here, the authors propose the identification of the longlived intermediate QD– state for exciton generation of CdSe-based QD-LEDs by a room temperature electrically-pumped single-molecule spectroscopy.

## Introduction

Colloidal semiconductor nanocrystals are an important class of solution-processable inorganic luminescent materials^1–5^. In recent decades, remarkable advances have been made in solution-processed light-emitting diodes (LEDs) based on colloidal nanocrystals, including CdSe-based quantum dots (QDs)^6–18^, InP-based QDs^19,20^, and perovskite nanocrystals^21,22^, paving the way towards a new generation of display and lighting technologies.

The emissive states for electroluminescence (EL) from QDs are excitons (bound electron–hole pairs). In-depth understanding of the generation and recombination of excitons is essential for interpreting device operation mechanisms and providing guidelines for future developments. Spectroscopic techniques have been applied to study the QD-LEDs^23–25^, revealing the recombination dynamics of the in-situ photo-generated excitons. In contrast, few attempts have been made to explore the exciton-generation processes in EL devices based on nanocrystals.

The current understanding of electrical excitation of nanocrystals is largely inherited from the conventional theories developed for bulk semiconductors^14,17^. A brief picture of this process is that electrons and holes are simultaneously diffused or injected into the emission zone, leading to populations of opposite-sign charge carriers statistically independent of each other^26^. However, distinctive from bulk inorganic semiconductors, nanocrystals show efficient radiative recombination of single-exciton states and even faster non-radiative Auger recombination of charged-exciton states or multi-exciton states because of the strong spatial confinement of charge carriers within individual nanocrystals^27,28^. In other words, high-efficiency nanocrystal-based EL requires efficient and balanced charge injection to generate single excitons in individual nanocrystals. Thus, it is of fundamental interest to go beyond the conventional “bulk interpretations” and elucidate the unique mechanism for exciton generation in nanocrystal-based EL devices.

Here we address a fundamental question of how the excitons are electrically generated in individual QDs. We develop room-temperature electrically pumped single-nanocrystal spectroscopy to reveal the hidden elementary processes and the associated kinetics at the single-nanocrystal level. The observations reveal a sequential electron–hole injection mechanism which is enhanced by charge confinement, an intrinsic property of nanocrystals. The unique nanoscopic mechanism is invoked to interpret efficient exciton generation in state-of-the-art QD-LEDs.

## Results

### Electrically pumped single-nanocrystal spectroscopy

We start with the electrical generation of excitons in an individual QD. An isolated CdSe-CdZnS core–shell QD (Supplementary Fig. 1) is used as an emitter in a single-dot EL device^29^. In this single-dot device (Supplementary Fig. 2), exciton migration and charge transport between neighbouring QDs^30–32^ are absent, providing an ideal system for investigating charge dynamics of exciton generation. The single CdSe-CdZnS QD in the EL device exhibits ideal photoluminescence (PL) properties, including single-channel radiative decay and stable single-exciton emission (Supplementary Fig. 3). When a forward DC bias of 1.85–2.30 V is applied, the single-QD EL device demonstrates background-free and stable single-exciton EL with emission rates of 10^3^–10^5^ s^−1^ (Supplementary Fig. 4).

**Fig. 1:**
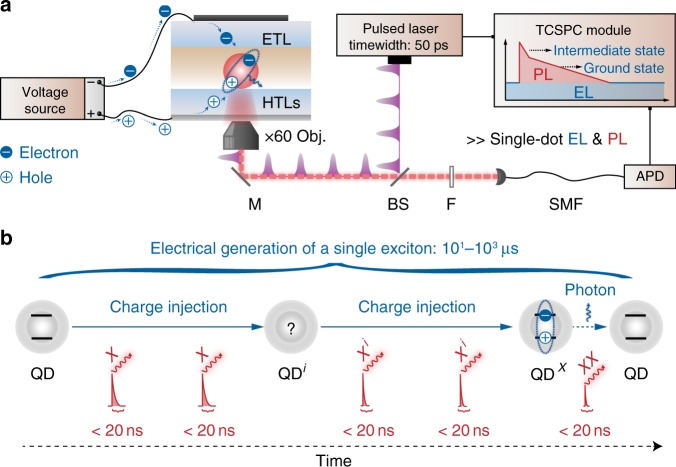
Electrically pumped single-nanocrystal spectroscopy. **a** Schematic diagram of the experimental setup. Electrical excitation (voltage source) and optical excitation (450 nm pulsed laser) are simultaneously applied on the single QD. Intensities and time-correlated single-photon-counting data are recorded simultaneously, enabling probe of states relevant to the single-dot EL. Optical excitation is kept in the single-exciton regime (average excitation per pulse <0.1) so that it does not alter the charging status of the single QD. **b** Temporal evolution of a single QD under electrical excitation. The pulsed laser serves as a fast and non-invasive probe. The average time of an EL cycle is substantially longer than the characteristic lifetimes of optically excited states. QD^i^ represents the possible intermediate states for single-dot EL processes (blue arrows). The corresponding optically excited states are denoted as X^i^ (in red).

We develop electrically pumped single-nanocrystal spectroscopy to probe the elementary charge-injection steps for the exciton generation in the single QD (Fig. 1a). At the single-dot level, the generation of a single exciton may consist of two possible pathways, i.e., the injection of one electron followed by the injection of one hole (${\mathrm{QD}}\mathop { \to }\limits^{{\mathrm{ + e}}} {\mathrm{QD}}^-\mathop { \to }\limits^{{\mathrm{ + h}}} {\mathrm{QD}}^{\mathrm{X}}$), and the injection of one hole followed by the injection of one electron (${\mathrm{QD}}\mathop { \to }\limits^{{\mathrm{ + h}}} {\mathrm{QD}}^ + \mathop { \to }\limits^{{\mathrm{ + e}}} {\mathrm{QD}}^{\mathrm{X}}$). The intermediate states of the two pathways are different, with one being negatively charged state (${\mathrm{QD}}^-$), and another one being positively charged state (${\mathrm{QD}}^ +$). The charging status of the CdSe-based QDs can be optically distinguished^33,34^. For the CdSe-CdZnS QD in the single-dot EL device, the lifetimes of the excited states of a neutral QD, a negatively charged QD, and a positively charged QD (denoted as X, X^−^ and X^+^, respectively) are determined to be 17.4 ± 2.6, 2.7 ± 0.3 and 1.6 ± 0.2 ns, respectively (Supplementary Fig. 5). The recombination processes of all optically excited states are much faster than the electrical generation of a single exciton (10–10^3^ µs, estimated from the emission rates of the single-dot EL device in the given voltage range). Therefore, we use a pulsed laser to excite the single QD under constant electrical pumping, allowing the probing of all possible states relevant to the single-dot EL (Fig. 1b). Note that the pulsed-laser excitation with a low power density (<0.1 excitation per pulse, 2.5 MHz) does not alter the charging status of the single QD, as demonstrated by the nonblinking PL intensity-time trace^35,36^ (Supplementary Fig. 3). In addition, the probability of the electrical injection of a charge carrier into an optically excited state is extremely low (<0.2%) because the lifetime of optically excited states in the single QD is very short relative to the time required for electrical excitation (see Methods for details). Hence, the optical excitation serves as a non-invasive probe to monitor the single-dot EL processes.

### Electrical generation of single excitons in a single QD

**Fig. 2:**
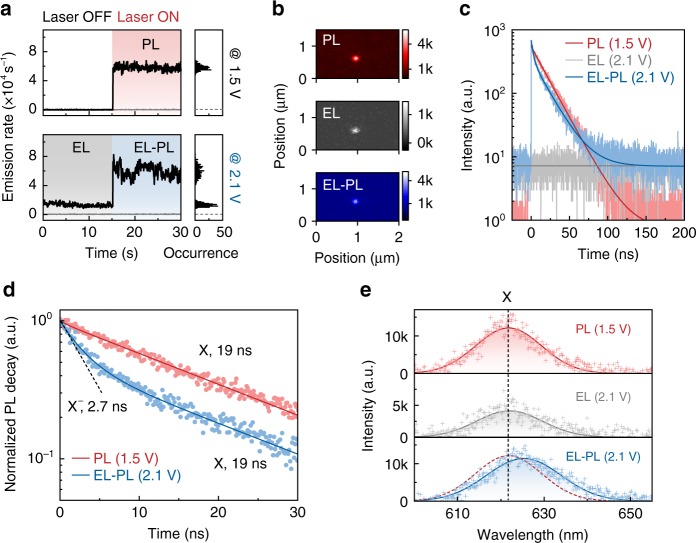
QD^−^ as the intermediate state for the electrical generation of a single exciton. **a** Intensity–time traces of a single QD under different excitation conditions. “PL” (red shaded regions), “EL” (grey-shaded regions) and “EL-PL” (blue shaded regions) traces correspond to emissions recorded under optical excitation, electrical excitation and simultaneous optical and electrical excitation, respectively. Grey lines are background counts. **b** Microscopy images of the single QD under different excitation conditions. **c** TCSPC data of PL (red), EL (grey) and EL–PL (blue) emissions. The data of EL emission (grey), which matches with the baseline of the EL–PL emission, is also shown. **d** PL decay for the single QD with electrical injection (blue) or without electrical injection (red), extracted from **c**. Solid lines are exponential fits and the dashed line is for visual guidance. **e** Single-dot spectra measured under different conditions. The single-exciton PL spectrum (red-dashed line) is also shown at the bottom for comparison.

A single QD is subject to three excitation conditions, i.e., pulsed-laser excitation, electrical excitation at a constant bias of 2.1 V, and simultaneous electrical and pulsed-laser excitations (Fig. 2a, b). The corresponding PL, EL, and EL–PL intensity–time traces (Fig. 2a) and time-correlated single-photon-counting (TCSPC) results (Fig. 2c) are recorded. Comparing with the stable PL and EL intensity–time traces (red and grey regions, Fig. 2a), intensity fluctuations occur in the EL–PL emission (blue region, Fig. 2a). This feature is attributed to electrical excitation-induced fast switching between charged states of the single QD within the bin time (50 ms)^37^. Regarding the TCSPC data of the EL–PL emission (blue curve in Fig. 2c), the EL contribution can be treated as the baseline because the continuous electrical excitation is independent of the pulsed optical excitation. The intensity of EL contribution in the EL–PL emission is identical to that of the EL emission (grey curve in Fig. 2c), confirming the negligible impact of optical excitation on the single-dot EL processes. This feature allows the extraction of the PL decay characteristics of the QD under electrical excitation from the EL–PL emission by subtracting the EL baseline (see Methods for details). The resultant curve (blue, Fig. 2d) is well-described by a double-exponential function. According to the characteristic decay dynamics (Supplementary Fig. 5), the fast component (2.7 ns) is assigned as X^−^, but not X^+^. The slow component (19 ns) is identical to the single-exciton decay measured without electrical injection (red curve in Fig. 2d). The fact that X^−^ contributes to the EL–PL emission is supported by spectral analyses. For the CdSe-CdZnS QD, the emission spectrum of X^−^ is featured by a redshift of 16 meV compared with that of X (Supplementary Fig. 6). As shown in Fig. 2e, the EL–PL spectrum shows broadening in the linewidth and redshift in the peak wavelength with respect to the spectrum of single-exciton emission. Therefore, the PL response of the QD under electrical excitation is composed of both X and X^−^, indicating that the exciton generation is mediated by a QD^−^ state.

Parallel measurements on 15 different single dots by electrically pumped single-nanocrystal spectroscopy (constant bias: 2.1 V) reveal the exclusive occurrences of X^−^, ruling out the generation of X^+^ (Supplementary Fig. 7). Furthermore, electrical excitation-dependent experiments show that only X and X^−^ contribute to the PL response of the single QD in the operating device with EL emission rates in the range of 21,000–70,000 s^−1^ (Supplementary Fig. 8). These results unambiguously demonstrate the exclusive existence of QD^−^ (rather than QD^+^) as the intermediate state, indicating that the generation of a single exciton follows the pathway of ${\mathrm{QD}}\mathop { \to }\limits^{{\mathrm{ + e}}} {\mathrm{QD}}^-\mathop { \to }\limits^{{\mathrm{ + h}}} {\mathrm{QD}}^{\mathrm{X}}$.

**Fig. 3:**
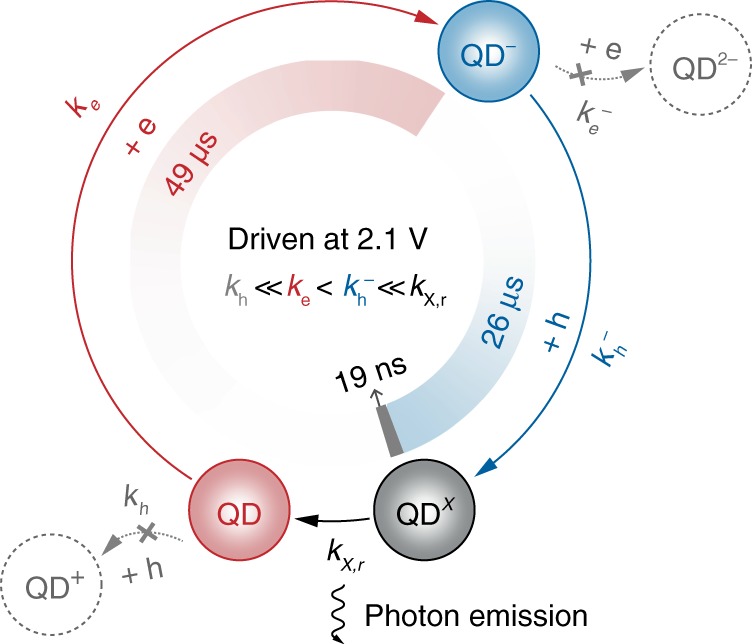
Dynamics of the single-dot EL cycle. EL of a single QD at 2.1 V consists of three elementary steps, i.e., electron injection into the neutral QD (red arrow, rate coefficient: $k_{\mathrm{e}}$ = 2.0 × 10^4^ s^−1^), hole injection into the negatively charged QD (blue arrow, rate coefficient: $k_{\mathrm{h}}^-$ = 3.8 × 10^4^ s^−1^) and radiative recombination of an exciton (black arrow, rate coefficient: $k_{{{\mathrm{X},{\mathrm{r}}}}}$ = 1/19 ns^−1^). At 2.1 V, hole injection into the neutral QD (${\mathrm{QD}}\mathop { \to }\limits^{{\mathrm{ + h}}} {\mathrm{QD}}^ +$) or electron injection into the negatively charged QD (${\mathrm{QD}}^-\mathop { \to }\limits^{{\mathrm{ + e}}} {\mathrm{QD}}^{{\mathrm{2-}}}$) is negligible according to spectroscopic results. Residence times of the three states, i.e., characteristic times of three elementary processes, are illustrated, indicating a long-lived intermediate state of ${\mathrm{QD}}^-$.

The spectroscopic results are used to analyse the dynamics of the elementary charge-injection steps (Fig. 3). When the single-dot EL device is biased at 2.1 V, the photon emission rate is estimated to be 1.33 × 10^4^ s^−1^, corresponding to a single-dot EL cycle of ~75 μs (${\mathrm{QD}}\mathop { \to }\limits^{{\mathrm{ + e}}} {\mathrm{QD}}^-\mathop { \to }\limits^{{\mathrm{ + h}}} {\mathrm{QD}}^{\mathrm{X}} \to {\mathrm{QD}}$). Given that the relative luminescent efficiency of X^−^ to X is ~25% (see the PL blinking trace shown in Supplementary Fig. 5), analyses on the PL decay properties of the EL–PL emission (Fig. 2d) reveals that the occupation probabilities of the ground state and the ${\mathrm{QD}}^-$ state in the EL cycle are ~65% and ~35%, respectively (see Methods for details). Accordingly, residence times of the ground state and the ${\mathrm{QD}}^-$ state in the EL cycle are determined to be ~49 and ~26 μs, respectively. The results suggest a long-lived intermediate state of ${\mathrm{QD}}^-$. The residence time of the ${\mathrm{QD}}^{\mathrm{X}}$ state, i.e., the single-exciton lifetime (~19 ns), is orders of magnitude shorter than the period of an EL cycle, accounting for the absence of PL responses from the electrically generated ${\mathrm{QD}}^{\mathrm{X}}$ (bi-exciton emission) in the EL–PL emission. The rate coefficients for the injection of one electron into the neutral QD ($k_{\mathrm{e}}$) and for the injection of one hole into the negatively charged QD ($k_{\mathrm{h}}^-$) are derived by using rate equations in the steady-state condition (see Methods for details). The results indicate that $k_{\mathrm{h}}^-$ (3.8 × 10^4^ s^−1^) is comparable to $k_{\mathrm{e}}$ (2.0 × 10^4^ s^−1^). Considering that no intermediate state of ${\mathrm{QD}}^{\mathrm{X}}$ is detected, the rate coefficient for the injection of one hole into the neutral QD (${\mathrm{QD}}\mathop { \to }\limits^{{\mathrm{ + h}}} {\mathrm{QD}}^ +$), $k_{\mathrm{h}}$, is negligible. Therefore, the rate coefficients of the electrical generation of a single exciton in the single-dot EL device (at a constant bias of 2.1 V) follow $k_{\mathrm{h}} \ll k_{\mathrm{e}}{\mathrm{ < }}k_{\mathrm{h}}^-$.

Charge dynamics of the single-dot EL cycle summarized in Fig. 3 suggests that confinement-enhanced Coulomb effects enable the generation of single excitons. In our single-dot EL device, the energy-level alignment of the oxide electron-transport layer, the neutral QD, and the polymeric hole-transport layer favours electron injection and hinders hole injection into a neutral QD (Supplementary Fig. 9), leading to $k_{\mathrm{h}} \ll k_{\mathrm{e}}$. The injection of one electron into a neutral QD results in a long-lived state of ${\mathrm{QD}}^-$. For CdSe-CdZnS QDs, the charge carriers are confined within the inner part of the QD (core radius: ~1.6 nm). The small self-capacitance of the nanocrystal induces Coulomb charging effects in the single QD^38–40^. From an energetic point of view, the addition of one electron shifts the electrical potential of the single QD toward higher energies (Supplementary Fig. 9). Hence, the rate for injection of a second charge carrier into the negatively charged QD is modulated by confinement-enhanced Coulomb interactions. Specifically, hole injection is significantly enhanced due to Coulomb attractive interaction, resulting in $k_{\mathrm{h}}^-$ substantially greater than $k_{\mathrm{h}}$. In consequence, single excitons can be efficiently generated at operating voltages close to the voltage corresponding to the optical bandgap of the QD (Supplementary Fig. 4). Furthermore, the fact that ${\mathrm{QD}}^{\mathrm{X}}$ is the dominating emissive state for the single-dot EL indicates injection of one electron and one hole into the QD in almost all single-dot EL cycles. Injection of a second electron into the negatively charged QD (${\mathrm{QD}}^-\mathop { \to }\limits^{{\mathrm{ + e}}} {\mathrm{QD}}^{{\mathrm{2-}}}$) is greatly suppressed due to repulsive Coulomb interaction.

### Electrical generation of single excitons in QD-LEDs

**Fig. 4:**
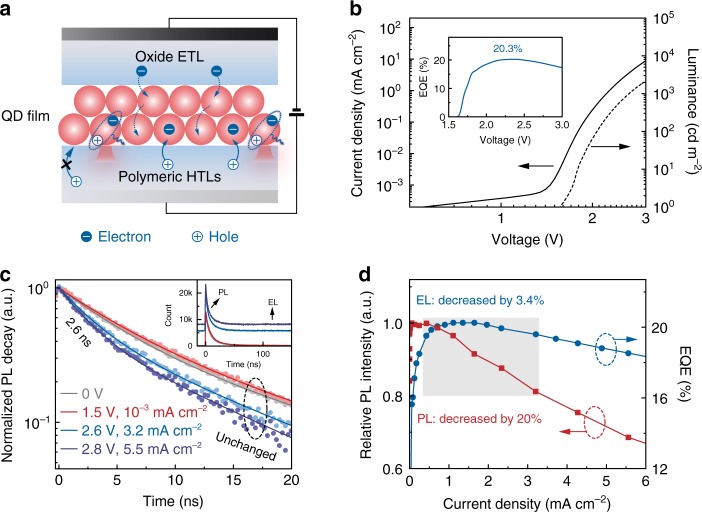
Exciton generation in a high-efficiency QD-LED. **a** Schematic diagram showing single-exciton generation via ${\mathrm{QD}}^-$ (${\mathrm{QD}}\mathop { \to }\limits^{{\mathrm{ + e}}} {\mathrm{QD}}^-\mathop { \to }\limits^{{\mathrm{ + h}}} {\mathrm{QD}}^{\mathrm{X}}$) in the QD-LED. Holes are injected into the negatively charged QDs close to the interface of hole-transport layer/QD layer. **b** Current density and luminance versus voltage characteristics. Inset: the EQE–voltage curve. **c** PL decay of the QD film in the working QD-LED driven at different voltages, measured by the microscopy system shown in Supplementary Fig. 11. Inset: the original TCSPC data showing baselines originated from EL emissions. In the experiments, photon-counting rates are reduced by using an extra neutral density filter to meet the requirement of the TCSPC technique. **d** EQE and relative PL intensity of the QD-LED at different current densities. The grey-shaded region corresponds to the high-efficiency regime, in which a significant discrepancy between changes in the EL and PL efficiencies is observed.

We propose that the mechanism of charge confinement-enhanced sequential electron–hole injection derived from EL of an individual QD can be invoked to interpret the exciton generation in the state-of-the-art QD-LEDs (Fig. 4a). We fabricate a red QD-LED comprised of an emissive film of the CdSe-CdZnS QDs (~2 monolayers) and similar charge-transport layers as in the single-dot EL device (Fig. 4a). This device exhibits a low turn-on voltage of 1.7 V (2.4 cd m^−2^) and a peak external quantum efficiency (EQE) of 20.3%, corresponding to an internal quantum efficiency of ~81% (Fig. 4b). These characteristics indicate the efficient generation of single excitons in the QD-LED. At the same time, a large energy barrier for hole injection into the neutral QDs is recognized, as reflected by the fact that the current densities of a hole-only device are approximately three orders of magnitude smaller than those of an electron-only device (Supplementary Fig. 10). For this QD-LED with asymmetric energy barriers for charge injection, a conventional scenario of exciton generation via independent charge injection (${\mathrm{QD}}\mathop { \to }\limits^{{\mathrm{ + e}}} {\mathrm{QD}}^-{\mathrm{;QD}}\mathop { \to }\limits^{{\mathrm{ + h}}} {\mathrm{QD}}^ +$) followed by a bi-molecular process (${\mathrm{QD}}^ + + {\mathrm{QD}}^- \to {\mathrm{QD}}^{\mathrm{X}} + {\mathrm{QD}}$) would predict unbalanced charge injection and thereby low efficiency, which is in contradiction to the device performance. Instead, we suggest that hole injection is assisted by confinement-enhanced Coulomb interactions. In other words, exciton generation in QD-LEDs relies on the charging of individual QDs (close to the hole-transport layer/emissive layer interface) with electrons, which in turn allows hole injection into the negatively charged QDs (Fig. 4a).

Our hypothesis is verified by in situ/operando optical measurements on the working QD-LED. TCSPC measurements on the QD film under electrical injection (Supplementary Fig. 11 for the experimental setup) show that an additional fast decay component becomes more apparent with increasing electrical-excitation levels, while the slow decay characteristics remain unchanged (Fig. 4c). Estimated from multi-exponential fitting of the curves, the lifetime of the emerged fast component (~2.6 ns) is distinctively different from the characteristic lifetime of X^+^ and resembles the characteristic lifetime of X^−^ acquired from single-dot spectroscopy, suggesting the occurrence of ${\mathrm{QD}}^-$ states. The fact that some of the QDs in the working device are negatively charged (with electrons) is also supported by the in situ measurements of relative PL intensity of the QD layer (see Supplementary Fig. 12 for the experimental setup). The results show that the PL efficiency of the QD layer decreases with the increase of current density (Fig. 4d). In the meantime, a significant discrepancy between the changes of PL and EL efficiencies at different current densities is observed. For example, when the current density increases from 0.4 to 3.2 mA cm^−2^ (shaded region in Fig. 4d), the relative PL intensity of the QD film shows a monotonic decrease of up to ~20% while the EQEs (in the range of 19.6–20.3%) exhibit minimal relative changes of ~3.4%. In this working regime, electric-field-induced effects, which would simultaneously reduce the EL and PL efficiencies^24,41^, are not the main causes responsible for the decrease of PL efficiency. The decrease of PL efficiency of the QD layer can be understood as a portion of QDs is charged with one electron, leading to ${\mathrm{QD}}^-$ states with a lower PL efficiency (~25%). The emergence of ${\mathrm{QD}}^-$ states does not necessarily cause non-radiative Auger recombination in the EL processes^23^. Instead, the negatively charged individual QDs favour hole injection accelerated by confinement-enhanced Coulomb interactions, allowing the efficient generation of neutral single excitons.

## Discussion

Our study unravels a nanoscopic picture of sequential electron-hole injection into individual CdSe-based QDs, which is enabled by the confinement-enhanced Coulomb effects, for the efficient generation of single excitons in QD-based EL devices. It is indeed interesting to see that at the single-nanocrystal level, despite that hole injection into a neutral QD is inefficient, the addition of an electron into the QD substantially increases the rate coefficient of hole injection to a level comparable to that of the efficient electron injection. The identification of the long-lived intermediate ${\mathrm{QD}}^-$ state for exciton generation shall provide a new lead on the mechanisms of efficiency loss and device degradation in state-of-the-art CdSe-based QD-LEDs. Furthermore, the electrically pumped single-nanocrystal spectroscopy developed in this work can be extended for the investigation of electrical-excitation processes in other types of nanocrystals, such as InP-based QDs and perovskite nanocrystals.

Considering that Coulomb interactions are pronounced in all nanocrystals with strong carrier confinement, we anticipate the charge confinement-enhanced efficient exciton generation to be a general mechanism for the nanocrystal-based EL devices, including LEDs, electrically driven single-photon sources^29^ and potentially lasers^42,43^. It would be possible to tailor the charge-confinement effects by engineering the size and dielectric properties of individual nanocrystals. Thus, our findings may lead to new approaches for modulating charge balance in nanocrystal-based EL devices.

## Methods

### Materials

Poly(methyl methacrylate) (PMMA, average molecular weight, ~120,000 g mol^−1^) and zinc acetate hydrate (>98%) were purchased from Sigma Aldrich. Poly(*N*,*N*′-bis(4-butylphenyl)-*N*,*N*′-bis(phenyl)-benzidine) (poly-TPD, average molecular weight, ~55,000 g mol^−1^) and poly(9,9-dioctylfluorene-co-*N*-(4-(3-methylpropyl))diphenylamine) (TFB, average molecular weight, ~50,000 g mol^−1^) were purchased from American Dye Source. Colloidal CdSe-CdZnS core–shell red QDs were purchased from Najing technology Co., Ltd. Tetramethylammonium hydroxide (TMAH, 98%) was purchased from Alfa-Aesar. Chlorobenzene (extra dry, 99.8%), octane (extra dry, >99%) and ethanol (extra dry, 99.5%) were purchased from Acros. Dimethyl sulfoxide (DMSO, HPLC grade) and ethyl acetate (HPLC grade) were purchased from J&K Chemical Ltd. ITO. Colloidal Zn_0.9_Mg_0.1_O nanocrystals were synthesized according to a previous report^44^.

### Device fabrication

The structure of the single-dot EL device is ITO (100 nm)/PEDOT:PSS (40 nm)/poly-TPD (30 nm)/single QDs covered by a PMMA layer (12 nm)/Zn_0.9_Mg_0.1_O (65 nm)/Al (100 nm). Single-dot EL devices were fabricated by depositing materials onto ITO coated glass slides (thickness: ~0.18 mm, resistance: ~50 Ω sq^−1^). PEDOT:PSS solutions (Baytron PVP Al 4083) were spin-coated onto the substrates at 3500 r.p.m. for 45 s and baked at 150 °C for 30 min. The PEDOT:PSS-coated substrates were subjected to an oxygen plasma for 4 min and then transferred to a nitrogen-filled glove box (O_2_ <1 ppm, H_2_O <1 ppm) for subsequent processes. Poly-TPD solutions (in chlorobenzene, 8 mg mL^−1^) were spin-coated at 2000 r.p.m. for 45 s and baked at 150 °C for 30 min. QD solutions (in octane, ~15 mg mL^−1^, diluted 20,000-folds before use), PMMA solutions (in acetone, 1.5 mg mL^−1^) and Zn_0.9_Mg_0.1_O nanocrystals (in ethanol, ~30 mg mL^−1^) were layer-by-layer spin-coated onto the substrates at 2000 r.p.m. for 45 s. Next, Al electrodes (100 nm) were deposited by a thermal evaporation system (Trovato 300 C) under high vacuum (~2 × 10^−7^ torr).

The structure of a QD-LED is ITO (100 nm)/PEDOT:PSS (40 nm)/TFB (45 nm)/QD (25 nm)/Zn_0.9_Mg_0.1_O (65 nm)/Ag (100 nm). QD-LEDs were fabricated by following procedures similar to those for the fabrication of single-dot EL devices. PEDOT:PSS solutions, TFB solutions (in chlorobenzene, 12 mg mL^−1^), QD solutions (in octane, ~15 mg mL^−1^) and Zn_0.9_Mg_0.1_O nanocrystals were spin-coated onto the ITO-coated glass substrates in sequence, and then Ag electrodes were deposited. Film deposition and post-processing parameters were the same as those for the fabrication of single-dot EL devices, except that the QD solutions were not further diluted for use.

The EL devices were encapsulated by using cover glass slides in a glove box.

### Characterizations of single QDs

Optical measurements of single QDs were conducted on a home-built fluorescence microscopic system equipped with a ×63 oil immersion objective (N.A. = 1.46) at room temperature (22–24 °C). The sample holder was a piezoelectric XYZ stage, allowing precise position control. A direct-voltage source (Keithley 2400) was used for electrical excitation and a laser diode (PicoQuant LHD-450) was used for optical excitation. A band-pass filter (621–643 nm, Semrock) was used to block the light from laser excitation. Microscopic images of emissions from single QDs were recorded by an EMCCD (Andor iXon3) with a 100 ms exposure time. Photons from a single QD were directed through a single-mode fibre pinhole and detected by single-photon avalanche photodetectors (SPCMs, PerkinElmer). Intensity traces and TCSPC data of a single QD were simultaneously recorded by a single-photon-counting module (PicoHarp 300) operating in the time-tagged time-resolved mode.

For electrically pumped single-nanocrystal spectroscopy measurements, the laser excitation was set at a low power density (~0.8 W cm^−2^, repetition rate: 2.5 MHz). During each measurement (Fig. 2a), a direct bias of 1.5 V (which does not generate EL), or 2.1 V (which generates EL), is applied to the device. The pulsed-laser excitation is off during the first 15 s and then turned on in the next 15 s. For optical excitation, the occupation probability of the optically excited states, i.e., the probability of finding the single QD at optically excited states, can be estimated from the product of the optical-excitation rate and the residence time of the optically excited state. Given the near-unity PLQY of the CdSe-CdZnS QDs, the optical-excitation rates can be estimated according to the corresponding PL emission rates. In our experiments, the optical-excitation rate is ~1 × 10^5^ s^−1^ and the lifetimes of all possible optically excited states of the QD are shorter than 20 ns, resulting in the occupation probability of the optically excited states less than 0.2%. Therefore, the probability of the electrical injection of a charge carrier into an optically excited state is less than 0.2%.

For measurements on single QDs in the PMMA matrix (Supplementary Fig. 5), the excitation level was mildly increased (~1.2 W cm^−2^, repetition rate: 1 MHz) to induce the dim states.

Second-order correlation functions of EL or PL from isolated QDs were measured by a Hanbury-Brown and Twiss setup consisting of a 50:50 beam splitter. Temporal intensities and time intervals of photons were simultaneously recorded by the single-photon-counting module, enabling the extraction of state-resolved second-order correlation functions.

### Characterizations of QD-LEDs

QD-LEDs were characterized under ambient conditions (room temperature: 22–24  °C and relative humidity: 40–60%). The current density–luminance–voltage (*J–L–V*) characteristics of the QD-LEDs were measured on a system consisting of a Keithley 2400 source meter and an integration sphere (Ocean Optics FOIS-1) coupled with a spectrometer (Ocean Optics QE-Pro). The devices were swept from zero bias to forward bias. The measurements were repeated many times with the peak EQEs and the efficiency roll-off curves unchanged.

In situ/operando measurements of transient PL of the QD film in the working QD-LED were conducted in the microscopic system shown in Supplementary Fig. 11. A ×60 water immersion objective (N.A. = 1.0) was used. The power density of the pulsed laser (450 nm, 2.5 MHz) was set to be ~8 mW cm^−2^, which corresponded to an optical excitation level of ~10^−3^ excitation per pulse for each QD. Electrical excitation was limited to a current density smaller than 5.6 mA cm^−2^, corresponding to an electrical injection rate <3 × 10^4^ s^−1^ (~30 µs per electrical excitation) for each QD. The experimental conditions ensured that the excitation levels were controlled in the single-exciton regime when optical excitation and electrical excitation were simultaneously applied. An extra neutral density filter was placed in front of the single-mode fibre. The photon-counting rate is two orders of magnitude smaller than the repetition rate of the pulsed laser, so that the requirement of TCSPC measurement is satisfied^45^.

For the measurements of relative PL intensity of QDs in operating QD-LEDs, a continuous 405 nm laser was used for optical excitation, and a direct-voltage source (Keithley 2400) was used for electrical excitation. The laser light was modulated at 1003 Hz by a chopper and focused on the operating device. The overall emission comprised of EL and modulated PL is collected by a photodetector (Thorlabs PDA100A). Signals from the photodetector were sent to a lock-in amplifier (SR830), where amplitudes of the ac-component of 1003 Hz, i.e. relative PL intensities, were extracted as the output. The power density of excitation light was kept at a low level to minimize influences of optical excitation on QD-LED operation.

### Other characterizations

The thickness measurements were conducted on atomic force microscopy (Asylum Research Cypher-S) in the tapping mode. The absorption spectra of QD solutions were measured by using a UV-Vis-NIR spectrophotometer (Agilent Cary-5000). The PL spectra were measured by a fluorescence spectrometer (Edinburgh Instruments FLS920). Transmission electron microscope images were obtained by using Hitachi 7700 operated at 80 keV.

### Transient PL analyses

PL decay curves are fitted with double-exponential functions written as

1 $$I_{t} = {{A}}_{{\mathrm{fast}}} \cdot {{e}}^{{\mathrm{-}}t/\tau _{{\mathrm{fast}}}} + {{A}}_{{\mathrm{slow}}} \cdot {{e}}^{{\mathrm{-}}t/\tau _{{\mathrm{slow}}}} + B,$$

where *I*_*t*_ is the transient light intensity. *τ*_fast_ and *τ*_slow_ are lifetimes of the two components. *B* represents background counts, which may include EL emissions. For all normalized PL decay curves, EL emissions are subtracted from the TCSPC data according to the fitting results. The fractional contribution of each component ($f_i$) is given by^45^

2 $$f_i = \frac{{A_i \tau _i}}{{{\sum} {A_i \tau _i} }}\,{\mathrm{and}}\, \, \,f_{{\mathrm{fast}}} + f_{{\mathrm{slow}}} = {\mathrm{1}}.$$

### Determination of the occupation probabilities

For a single QD switching between the neutral ground state (“QD” state) and the negatively charged state (“${\mathrm{QD}}^-$” state), fractional contributions of the two components in overall PL responses are connected to occupation probabilities of the corresponding states (denoted as $P_{{\mathrm{QD}}}$ and $P_{{\mathrm{QD}}^-}$, respectively) by the following equations:

3 $$f_{{\mathrm{slow}}} = \frac{{P_{{\mathrm{QD}}} \cdot {\mathrm{QY}}_{\mathrm{X}}}}{{P_{{\mathrm{QD}}} \cdot {\mathrm{QY}}_{\mathrm{X}} + P_{{\mathrm{QD}}^-} \cdot {\mathrm{QY}}_{\mathrm{X}^{-}}}}$$

4 $$f_{{\mathrm{fast}}} = \frac{{P_{{\mathrm{QD}}^-} \cdot {\mathrm{QY}}_{\mathrm{X}^{-}}}}{{P_{{\mathrm{QD}}} \cdot {\mathrm{QY}}_{\mathrm{X}} + P_{{\mathrm{QD}}^-} \cdot {\mathrm{QY}}_{\mathrm{X}^{-}}}}$$

where ${\mathrm{QY}}_{\mathrm{X}}$ and ${\mathrm{QY}}_{\mathrm{X}^{-}}$ are the PL QYs of X and X^−^, respectively. According to Supplementary Fig. 5, the relative PL QY of X^−^, i.e., ${\mathrm{QY}}_{\mathrm{X}^{-}}/{\mathrm{QY}}_{\mathrm{X}}$, is estimated to be 25%.

Given that $P_{{\mathrm{QD}}}{\mathrm{ + }}P_{{\mathrm{QD}}^-} \approx {\mathrm{1}}$, occupation probabilities of the two states are derived from transient PL results:

5 $$P_{{\mathrm{QD}}} = \frac{{f_{{\mathrm{slow}}}}}{\frac{{{\mathrm{QY}}_{\mathrm{X}}}}{{{\mathrm{QY}}_{\mathrm{X}^{-}}}} \cdot f_{{\mathrm{fast}}} + f_{{\mathrm{slow}}}},$$

6 $$P_{{\mathrm{QD}}^-} = \frac{{f_{{\mathrm{fast}}}}}{{f_{{\mathrm{fast}}} + \frac{{{\mathrm{QY}}_{\mathrm{{X}}^-}}}{{{\mathrm{QY}}_{\mathrm{X}}}} \cdot f_{{\mathrm{slow}}}}}.$$

### Rate-equation analyses on the single-dot EL cycle

The single-dot EL cycle (${\mathrm{QD}}\mathop { \to }\limits^{{\mathrm{ + e}}} {\mathrm{QD}}^-\mathop { \to }\limits^{{\mathrm{ + h}}} {\mathrm{QD}}^{\mathrm{X}} \to {\mathrm{QD}}$) can be described by rate equations associated with the transitions between the three states. The transition rates for the elementary steps are products of the corresponding rate coefficients ($k_i$) and the occupation probabilities of reactants ($P_i$). The rate coefficients for injection of one electron into the neutral QD (${\mathrm{QD}}\mathop { \to }\limits^{{\mathrm{ + e}}} {\mathrm{QD}}^-$) and for injection of one hole into the negatively charged QD (${\mathrm{QD}}^-\mathop { \to }\limits^{{\mathrm{ + h}}} {\mathrm{QD}}^ +$) are $k_{\mathrm{e}}$ and $k_{\mathrm{h}}^-$, respectively. The rate coefficient of single-exciton recombination (${\mathrm{QD}}^{\mathrm{X}} \to {\mathrm{QD}}$) is $k_{{{\mathrm{X},{\mathrm{r}}}}}$, which is the reciprocal of the single-exciton PL lifetime. We connect the occupation probabilities of the three states by the following rate equations:

7 $$\frac{{{\mathrm{d}}P_{{\mathrm{QD}}}}}{{{\mathrm{d}}t}} = -k_{\mathrm{e}} \cdot P_{{\mathrm{QD}}} + k_{{{{\mathrm{X}},{\mathrm{r}}}}} \cdot P_{{\mathrm{QD}}^{\mathrm{X}}}$$

8 $$\frac{{{\mathrm{d}}P_{{\mathrm{QD}}^-}}}{{{\mathrm{d}}t}} = k_{\mathrm{e}} \cdot P_{{\mathrm{QD}}}- k_{\mathrm{h}}^{-} \cdot P_{{\mathrm{QD}}^{-}}$$

9 $$\frac{ {\mathrm{d}} P_{{{\mathrm{QD}}^{\mathrm{X}}}} } {{{\mathrm{d}}t}} = k_{\mathrm{h}}^{-} \cdot P_{{{\mathrm{QD}}^{-}}}-k_{{{\mathrm{X}},{\mathrm{r}}}} \cdot P_{{{\mathrm{QD}}^{\mathrm{X}}}}$$

For the CdSe-CdZnS QDs with a near-unity PLQY, the photon emission rate of a single-dot EL device ($I_{{\mathrm{EL}}}$) is directly connected to the recombination rate of ${\mathrm{QD}}^{\mathrm{X}}$: $I_{{\mathrm{EL}}} = k_{{{{\mathrm{X}},{\mathrm{r}}}}} \cdot P_{{\mathrm{QD}}^{\mathrm{X}}}$.

In the steady condition of the single-dot EL cycle (${\mathrm{d}}P_{{\mathrm{QD}}}$/d*t* = d$P_{{\mathrm{QD}}^-}$/d*t* = d$P_{{\mathrm{QD}}^{\mathrm{X}}}$/d*t* = 0), we obtain

10 $$k_{{\mathrm{e}}} \cdot P_{{\mathrm{QD}}} = k_{{\mathrm{h}}}^{-} \cdot P_{{\mathrm{QD}}^{-}} = k_{{\mathrm{X}},{\mathrm{r}}} \cdot P_{{\mathrm{QD}}^{\mathrm{X}}} = I_{{\mathrm{EL}}}.$$

Accordingly, the injection-rate coefficients can be derived from the photon emission rate and the occupation probabilities of QD state and ${\mathrm{QD}}^-$ state by the following equations:

11 $$k_{\mathrm{e}} = \frac{{I_{{\mathrm{EL}}}}}{{P_{{\mathrm{QD}}}}} = \frac{1}{{\tau _{{\mathrm{QD}}}}},$$

12 $$k_{\mathrm{h}}^{-} = \frac{{I_{{\mathrm{EL}}}}}{{P_{{\mathrm{QD}}^-}}} = \frac{1}{{\tau _{{\mathrm{QD}}^-}}},$$

where $\tau _{{\mathrm{QD}}}$ and $\tau _{{\mathrm{QD}}^-}$ are characteristic times of electron injection and hole injection, respectively, which can be regarded as the residence times of QD state and ${\mathrm{QD}}^-$ state.

Considering an overall detection efficiency of ~10%, $I_{{\mathrm{EL}}}$ of a single QD under 2.1 V is estimated to be 1.33 × 10^4^ s^−1^. $P_{{\mathrm{QD}}}$ and $P_{{\mathrm{QD}}^-}$ are determined to be 65% and 35%, respectively. According to Eqs. (11) and (12), these values lead to $k_{\mathrm{e}}$ =  2.0 × 10^4^ s^−1^ ($\tau _{{\mathrm{QD}}}$: 49 μs) and $k_{\mathrm{h}}^-$ = 3.8 × 10^4^ s^−1^ ($\tau _{{\mathrm{QD}}^-}$: 26 μs), respectively.

## Supplementary information

Supplementary Information

## Data Availability

The data that support the finding of this study are available from the corresponding author upon reasonable request. The source data underlying Supplementary Fig. 5e and Supplementary Fig. 7 are provided as a Source Data file.

## Source Data

Source Data
