# Supplementary material for: Deciphering exciton-generation processes in quantum-dot electroluminescence
Source: Nat Commun. 2020 May 8;11:2309. doi: 10.1038/s41467-020-15944-z (PMC7210259; doi:10.1038/s41467-020-15944-z)
Supplement: Supplementary file 1 — Supplementary Information [file 41467_2020_15944_MOESM1_ESM.pdf]

Supplementary Information For

Deciphering exciton-generation processes in quantum-dot electroluminescence

Deng *et al.*

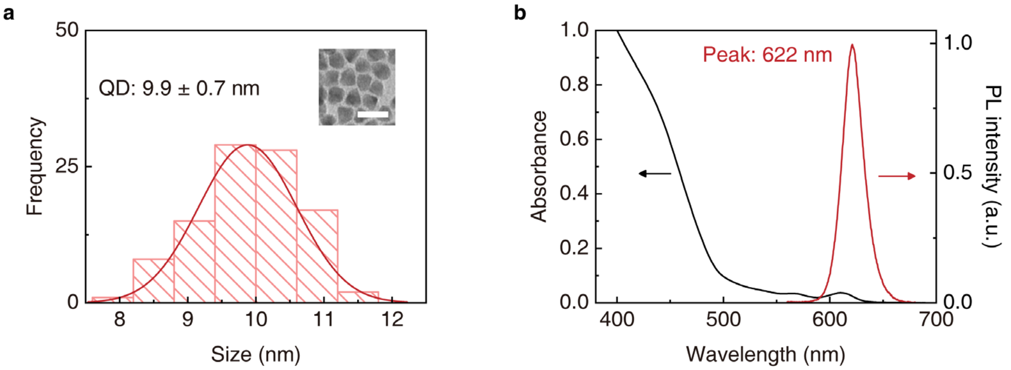

Supplementary Figure 1: The CdSe-CdZnS QDs. a Size distribution histogram of the CdSe-CdZnS QDs. Inset: a typical TEM image (scale bar: 20 nm). **b** Ultraviolet-visible absorption and PL spectra of the solution of QDs in octane.

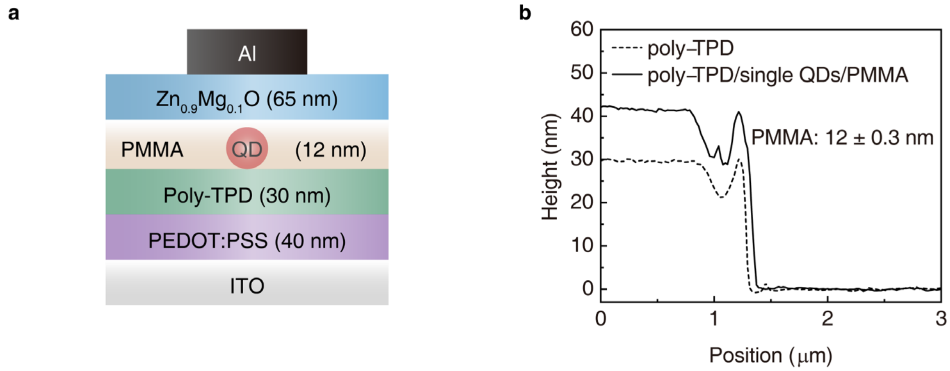

Supplementary Figure 2: A single-dot EL device. a Device structure: ITO/PEDOT:PSS/poly-TPD/Single QD in PMMA/$Zn_{0.9}Mg_{0.1}O$/Al. The isolated QDs are deposited onto the polymeric hole-transport layers and encapsulated by a thin layer of PMMA. The $Zn_{0.9}Mg_{0.1}O$ nanocrystals are used as an electron-transport layer. **b** Thickness measurement of the PMMA layer. Cross-section profiles are measured by an atomic force microscopy in the tapping mode. Results demonstrate a flat surface (solid line, position: 0–0.7 μm) after the deposition of the sparsely dispersed single QDs and the PMMA layer onto the poly-TPD film. The overall thickness of the PMMA layer (12 ± 0.3 nm) is slightly larger than the average diameter of the CdSe-CdZnS QDs (9.9 ± 0.7 nm), suggesting that the single QDs are immersed into the PMMA layer (as illustrated in the device structure). Note that the trenches (position: 0.8–1.3 μm) are caused by scratching procedures to create the steps required for the thickness measurements.

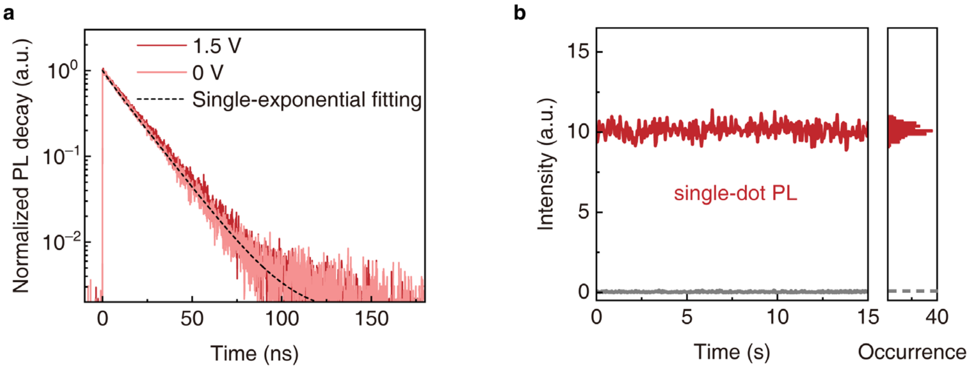

Supplementary Figure 3: PL properties of a single QD in the single-dot EL device. a PL decay curves of a single QD measured at 0 V and 1.5 V (below the turn-on threshold, ~1.85 V). The two curves are nearly identical and both curves can be fitted by a single-exponential function with a characteristic lifetime of 19 ns (dashed line). The results suggest negligible electric-field-induced effects for the QD in the single-dot EL device. Background PL emission from the hole-transport layer (poly-TPD) in the device (contribution < 5%) was subtracted. **b** PL intensity-trace of a single QD in the device and the corresponding histogram. The grey lines are background baselines. The non-blinking feature, i.e., no switching between states with different PL efficiencies, suggests that photo-induced ionization processes are negligible. These properties indicate that the single QD in the single-dot EL device offers stable single-exciton emission under our optical-excitation conditions.

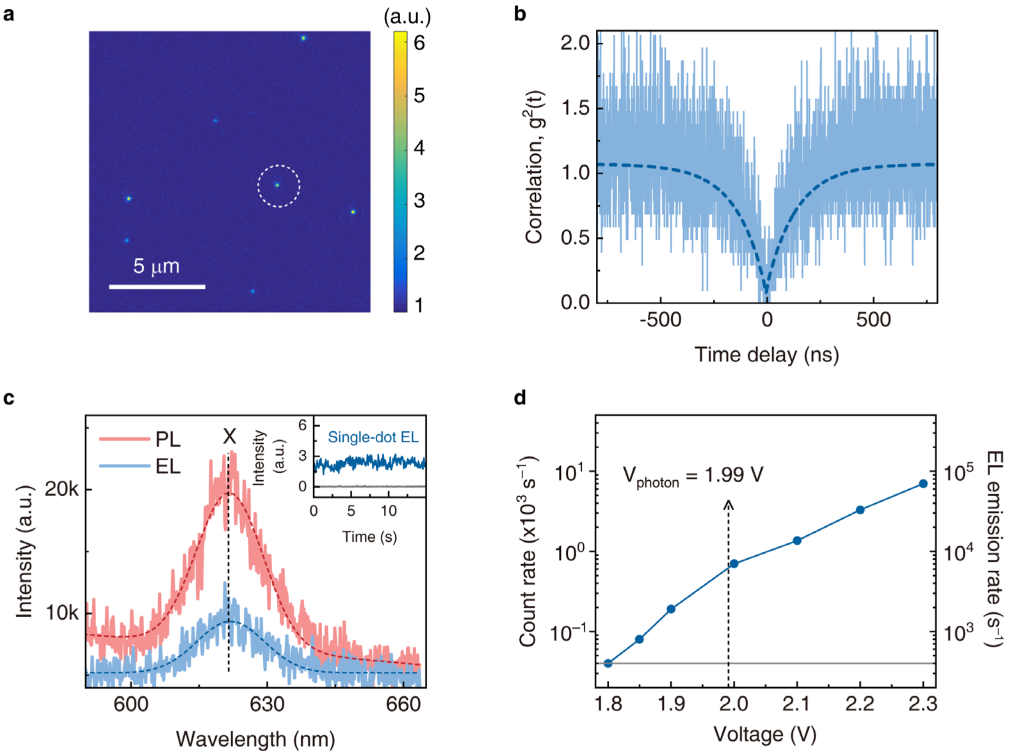

Supplementary Figure 4: EL behaviours of the single-dot device. a Microscopic image of EL from isolated single QDs (driven at 2.15 V). Scale bar: 5 µm. **b** A second-order correlation function ($g^2(t)$, blue line) of single-dot EL (driven at 2.15 V). The value of $g^2(0)$ is determined to be 0.07 by exponential fitting (blue dashed line), indicating minimal bi-exciton emission in the single-dot EL[1, 2]. **c** PL (red) and EL (blue, driven at 2.1 V) spectra of a single QD share the same peak wavelength of 622 nm and an identical full width at half maximum (FWHM) of 18 nm (integration time: 4 s). Inset: An EL intensity-time trace of the single QD showing stable single-exciton emission. The emission on the short wavelength side of the PL spectrum originates from PL of the hole-transporting layer, poly-TPD. The absence of background emission from poly-TPD in the EL spectrum suggests the negligible leakage current. Dashed lines are fits to the data. **d** Voltage-dependent EL intensity of a single QD (circled in **a**). The internal EL emission rates (right) are derived from the photon-counting rates (left) according to an overall detection efficiency of ~10%. The background counts are illustrated by the grey line. The threshold voltage for the single-dot EL is ~1.85 V, which is below the voltage (dashed black arrow) corresponding to the bandgap of the QD (1.99 eV).

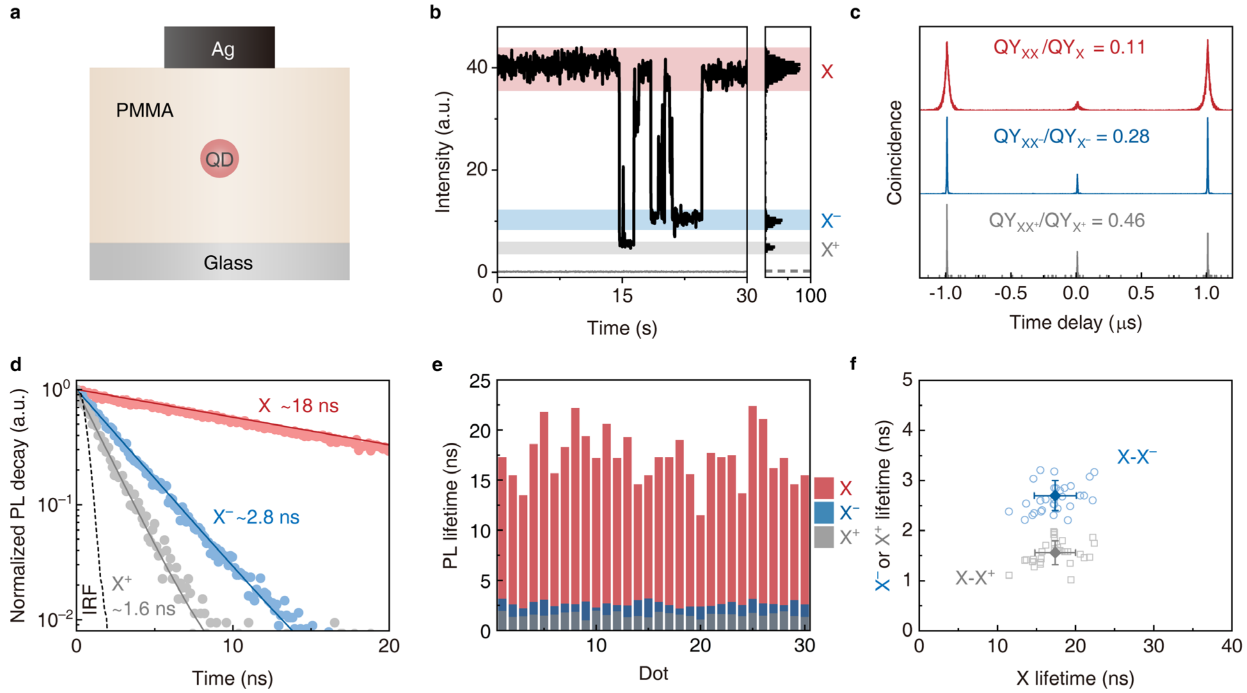

Supplementary Figure 5: Optical properties of X, X^- and X^+ of a single CdSe-CdZnS QD in the presence of a metal electrode. a Single QDs were dispersed in a PMMA matrix (thickness ~100 nm) by spin-coating a solution of QDs and PMMA in toluene (3:100 by weight) onto a glass slide, followed by deposition of a silver electrode. **b** Representative PL intensity-time trace of a single QD (measured under pulsed laser excitation) and the corresponding histogram showing switching between three emissive states. For CdSe-based QDs, the "bright state" (red shaded), the "dim state" with a higher QY (blue shaded) and the "dim state" with a lower QY (grey shaded) are assigned as X, X^- and X^+, respectively. $g^2(t)$ curves and PL decay curves for the three states can be selectively extracted from the corresponding intensity-windows. **c** $g^2(t)$ curves for X (red), X^- (blue) and X^+ (grey) of the single QD extracted according to the temporal intensities. The PL QY ratios of bi-exciton to single exciton are derived by following an established method[3]. According to a recent report[4], QY_{XX^-}/QY_{X^-} is smaller than QY_{XX^+}/QY_{X^+} for CdSe-based QDs because of the different radiative and Auger recombination pathways of X^- and X^+. Thus, the results confirm our assignments of X^- and X^+. **d** PL decay curves of for X (red), X^- (blue) and X^+ (grey) of the single QD extracted according to the temporal intensities. The solid lines are the single-exponential fitted curves. The grey dashed curve represents the instrument response function (0.78 ns). **e** PL lifetimes of X (red), X^- (blue) and X^+ (black) of 30 single QDs. **f** The corresponding statistical

distribution of PL lifetimes. Blue squares and grey circles represent lifetime correlations of X-X^- and X-X^+ for each QD, respectively. Average values and the corresponding standard deviations of the PL lifetimes are illustrated by diamonds with error bars. The characteristic lifetimes of X, X^- and X^+ are determined to be 17.4 ± 2.6 ns, 2.7 ± 0.3 ns, and 1.6 ± 0.2 ns, respectively. We note that in the single-dot EL device, the lifetime of X is decreased due to the electrode-induced Purcell effects[5]. Here, the photonic environment of the single QDs in the single-dot EL device is mimicked by the structure shown in **a**. The average X lifetime (17.4 ns) for the QDs agrees well with that in single-dot EL devices (17–19 ns), validating the similarity of the two photonic environments. Therefore, statistical results for X^- and X^+ displayed in **f** are used to identify charged states of single QDs in EL devices. The source data of lifetime distributions are provided as a Source Data file.

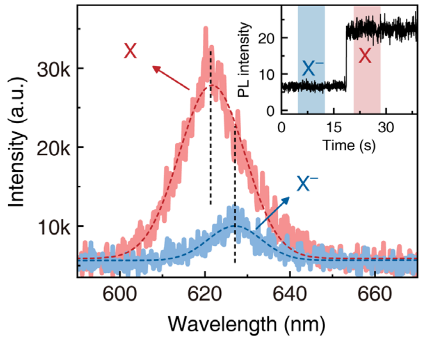

Supplementary Figure 6: PL spectra of X (red) and X⁻ (blue) of a single CdSe-CdZnS QD. The QD was incorporated into the structure shown in Supplementary Figure 5a. PL from a single QD was split by a 50:50 beam splitter and collected by two optical fibres. One output was sent to a spectrometer to record the single QD spectra, and another output was detected by an APD to monitor temporal intensities (inset). In this way, during the intensity blinking of a single QD, spectra of long "bright" durations corresponding to X emission, and spectra of long "dim" durations corresponding to X⁻ emission (PL intensity: ~25 % of "bright" states) were selectively recorded (integration time: 4 s). The peak of X⁻ emission is red-shifted by 5 nm in the wavelength (16 meV in photon energy) comparing with that of X emission. Dashed lines are fits to the data.

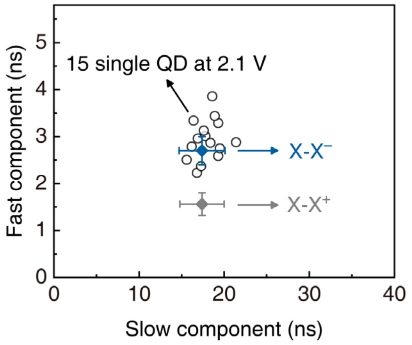

Supplementary Figure 7: Electrically-pumped single-nanocrystal spectroscopy on 15 different single QDs. Lifetimes (circles) of slow components (18.0 ± 1.5 ns) and fast components (2.9 ± 0.4 ns) are in agreement with characteristic lifetimes of X and X^- (blue diamond with the error bar, obtained from Supplementary Figure 5). The significant deviation from the characteristic lifetime of X^+ (grey diamond with the error bar, obtained from Supplementary Figure 5) excludes the possibility of QD^+ as the intermediate state. Error bars represent the corresponding standard deviations. The source data of lifetime distributions are provided as a Source Data file.

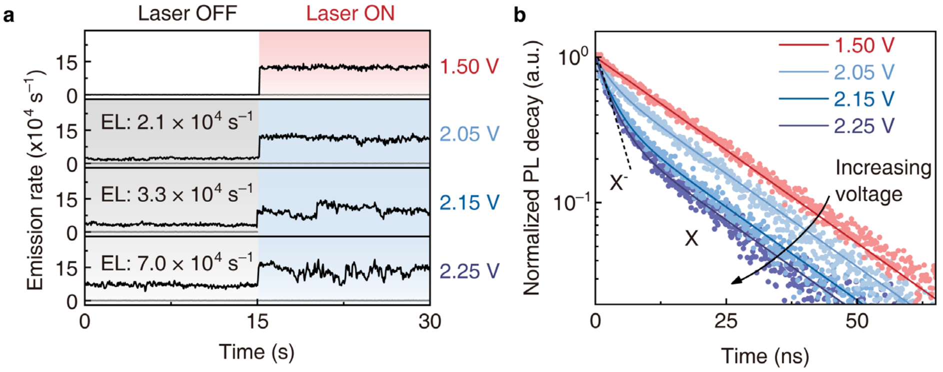

Supplementary Figure 8: Electrically-pumped single-nanocrystal spectroscopy on a single QD under different electrical-injection levels. a Intensity-time traces of a single QD under optical excitation (red shaded region), electrical excitation (grey shaded regions) and simultaneous optical and electrical excitation (blue shaded regions). The EL emission rates increase at higher voltages, leading to stable emission of up to ~7×10^4 s^{-1}. **b** Normalized PL decay curves of the single QD under different voltages (blue lines). PL decay of the single QD without electrical injection (red lines, measured at 1.50 V) is shown for comparison. All curves can be well fitted by double-exponential functions with fixed components of X^- (2.9 ns) and X (17 ns), as are shown by the solid lines. The fractional contributions of X^- become greater as the electrical injection level increases. Dashed lines are fits to the data.

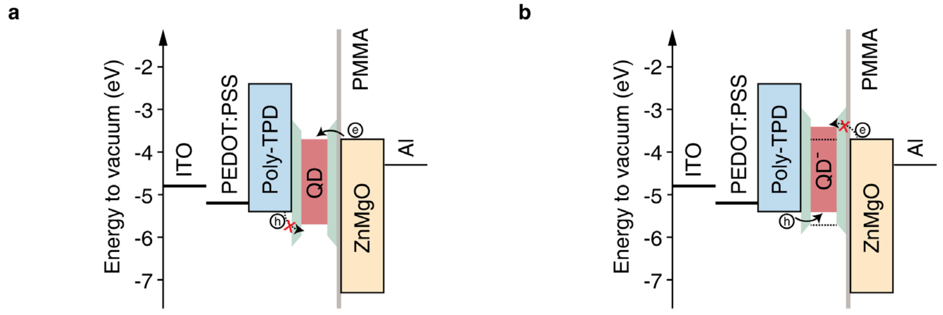

Supplementary Figure 9: Energy-level diagram of a single-dot EL device. a Schematic diagram for charge injection into a neutral QD. From an energetical point of view, electron injection (solid arrow) is favoured while hole injection (dashed arrow) is hindered due to the considerable offset between HOMO level of poly-TPD and the valance-band level of the CdSe-CdZnS QD. **b** Schematic diagram for charge injection into a negatively-charged QD. Due to Coulomb charging effects, the addition of one electron can shift the electrical potential of the single QD toward higher energy[6]. According to a classic electrostatic model[7], the upward energy shift (ΔE_c), i.e., the single-electron charging energy, is calculated from the capacitance of the QD (C) by the equation of $\Delta E_c = e^2/2C = e^2/(8\pi\varepsilon_0\varepsilon_r r)$. In the equation, e, r, and ε_r are the elementary electronic charge, the core radius of the QD, and the relative dielectric constant of the surrounding medium, respectively. For our QDs with a core radius of ~1.6 nm, the energy shift is estimated to be ~150 meV. This value agrees reasonably well with the charging energy measured from CdSe QDs with similar diameters[8]. Consequently, the hole-injection energy barrier for hole injection is reduced and the electron-injection energy barrier is increased.

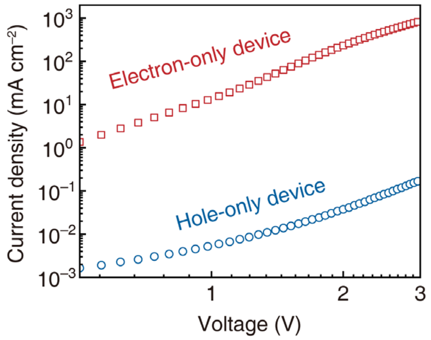

Supplementary Figure 10: Current densities versus voltages of an electron-only device and a hole-only device. The structure of the electron-only device is ITO/QDs (25 nm)/$Zn_{0.9}Mg_{0.1}O$ (65 nm)/Ag. The structure of the hole-only device is ITO/PEDOT:PSS (40 nm)/TFB (45 nm)/QDs (25 nm)/MoO_3 (5 nm)/Au.

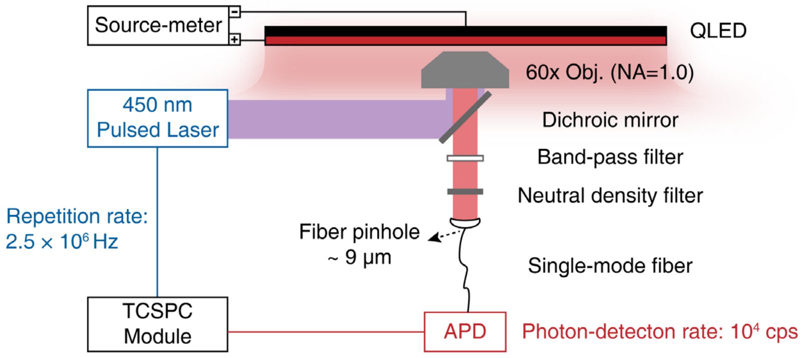

Supplementary Figure 11: Transient PL measurements on the working QD-LED. The experimental setup shares the same microscopic system shown in Fig. 1. The QD film was under simultaneous electrical excitation by a DC bias and optical excitation by a pulsed laser (450 nm, 2.5 MHz). An optical fibre (core size: ~9 μm) was used to collect the emissions from a micro area of the device. PL decay curves are extracted by subtracting the EL emissions from the micro area of the working QD-LED. We highlight that an extra neutral density filter is placed in front of the single-mode fibre so that the requirement of TCSPC measurement (the photon-counting rate is two orders of magnitude smaller than the repetition rate of the pulsed laser) is satisfied[9]. In addition, both optical and electrical excitation are controlled in the single-exciton regime (see the Methods section for details of the excitation conditions) to ensure that optical excitation and electrical excitation are independent of each other.

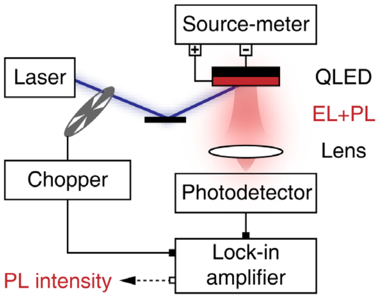

Supplementary Figure 12: In-situ measurements of relative PL intensity of the QD film in the working QD-LED. A frequency-modulated (1,003 Hz) low-intensity optical excitation (405 nm) is applied to the operating QD-LED. The relative PL intensities of the QD film are resolved by a lock-in amplifier.

Supplementary References

1. Lounis, B. et al. Photon antibunching in single CdSe/ZnS quantum dot fluorescence. *Chem. Phys. Lett.* **329,** 399–404 (2000).
2. Lin, X. et al. Electrically-driven single-photon sources based on colloidal quantum dots with near-optimal antibunching at room temperature. *Nat. Commun.* **8,** 1132 (2017).
3. Nair, G., Zhao, J. & Bawendi, M. G. Biexciton quantum yield of single semiconductor nanocrystals from photon statistics. *Nano Lett.* **11,** 1136–1140 (2011).
4. Xu, W. et al. Deciphering charging status, absolute quantum efficiency, and absorption cross section of multicarrier states in single colloidal quantum dots. *Nano Lett.* **17,** 7487–7493 (2017).
5. Barnes, W. L. Fluorescence near interfaces: the role of photonic mode density. *J. Mod. Opt.* **45,** 661–699 (1998).
6. Talapin, D. V., Lee, J.-S., Kovalenko, M. V. & Shevchenko, E. V. Prospects of colloidal nanocrystals for electronic and optoelectronic applications. *Chem. Rev.* **110,** 389–458 (2010).
7. Zabet-Khosousi, A. & Dhirani, A.-A. Charge transport in nanoparticle assemblies. *Chem. Rev.* **108,** 4072–4124 (2008).
8. Alperson, B., Cohen, S., Rubinstein, I. & Hodes, G. Room-temperature conductance spectroscopy of CdSe quantum dots using a modified scanning force microscope. *Phys. Rev. B* **52,** R17017–R17020 (1995).
9. Lakowicz, J. R. *Principles of Fluorescence Spectroscopy* (Springer, New York, 2006).
